# Self-Rated Health In Mongolian Older Adults: Insights From Childhood And Life-Course Factors

**DOI:** 10.1093/geroni/igaf122.4188

**Published:** 2025-12-31

**Authors:** Sugarmaa Myagmarjav, Zoljargalan Gantumur, Ariunsanaa Bagaajav, Saranchuluun Otgon

**Affiliations:** Mongolian National University of Medical Sciences, Ulaanbaatar, Ulaanbaatar, Mongolia; Virginia Commonwealth University, Richmond, Virginia, United States; City University of New York, Astoria, New York, United States; Mongolian National University of Medical Sciences, Ulaanbaatar, Ulaanbaatar, Mongolia

## Abstract

An upper-middle-income country in Asia, Mongolia, is experiencing rapid population aging, with the population aged 65 and above projected to triple by 2060. Healthy aging reflects socioeconomic, and health conditions accumulated across the life course rather than being determined only in old age. Recently, life-course factors have increasingly been emphasized in aging studies to understanding healthy aging better. A cross-sectional survey among 149 older adults aged 60 and above in Ulaanbaatar was carried out from January to March, 2025 to examine whether self-rated health across childhood, midlife, and later life is associated with socioeconomic conditions. A single-item question was used to measure self-rated health both in childhood and midlife. Life-course socioeconomic indicators included childhood (family financial status, parental income adequacy), midlife (education, occupation, salary satisfaction), and older age (pension, household income adequacy). Binary logistic regression examined associations between life-course factors and current health. Participants’ mean age was 68.8 ± 7.1 years, 40.3% were male, 61.8% married, and 57.7% urban residents. Overall, 43% reported poor health, with women more likely to report poor self-rated health than men. Childhood self-rated health was the only significant predictor of current health. Rural residence was also linked to poorer health, while education, marital status, and income showed no significant effects. Childhood health was a key predictor of current self-rated health. This study underscores the need to conduct future research with larger samples to validate the current results and identify additional life-course determinants of healthy aging.

